# Effects of climate warming on net primary productivity in China during 1961–2010

**DOI:** 10.1002/ece3.3029

**Published:** 2017-07-22

**Authors:** Fengxue Gu, Yuandong Zhang, Mei Huang, Bo Tao, Rui Guo, Changrong Yan

**Affiliations:** ^1^ Key Laboratory of Dryland Agriculture Ministry of Agriculture Institute of Environment and Sustainable Development in Agriculture Chinese Academy of Agricultural Sciences Beijing China; ^2^ Key Laboratory of Forest Ecology and Environment State Forestry Administration Institute of Forest Ecology, Environment and Protection Chinese Academy of Forestry Beijing China; ^3^ Key Laboratory of Ecosystem Network Observation and Modeling Institute of Geographical Sciences and Natural Resources Research Chinese Academy of Sciences Beijing China; ^4^ Department of Plant and Soil Sciences College of Agriculture, Food and Environment University of Kentucky Lexington KY USA

**Keywords:** Carbon Exchange between Vegetation, Soil and Atmosphere (CEVSA), climate warming, ecosystem modeling, net primary productivity

## Abstract

The response of ecosystems to different magnitudes of climate warming and corresponding precipitation changes during the last few decades may provide an important reference for predicting the magnitude and trajectory of net primary productivity (NPP) in the future. In this study, a process‐based ecosystem model, Carbon Exchange between Vegetation, Soil and Atmosphere (CEVSA), was used to investigate the response of NPP to warming at both national and subregional scales during 1961–2010. The results suggest that a 1.3°C increase in temperature stimulated the positive changing trend in NPP at national scale during the past 50 years. Regardless of the magnitude of temperature increase, warming enhanced the increase in NPP; however, the positive trend of NPP decreased when warming exceeded 2°C. The largest increase in NPP was found in regions where temperature increased by 1–2°C, and this rate of increase also contributed the most to the total increase in NPP in China's terrestrial ecosystems. Decreasing precipitation depressed the positive trend in NPP that was stimulated by warming. In northern China, warming depressed the increasing trend of NPP and warming that was accompanied by decreasing precipitation led to negative changing trends in NPP in large parts of northern China, especially when warming exceeded 2°C. However, warming stimulated the increase in NPP until warming was greater than 2°C, and decreased precipitation helped to increase the NPP in southern China.

## 1 INTRODUCTION

Air temperatures have increased by as much as 0.85°C [0.65–1.06°C] from 1880 to 2012, and they are predicted to rise another 0.3–4.8°C by 2081–2100 relative to 1986–2005, based on various modeled scenarios (International Panel on Climate Change (IPCC), 2014). A maximum reasonable increase in temperature of 2°C was first proposed by Nordhaus (1977). The Ministerial Conference of the European Union in 1996 first accepted the maximum threshold of 2°C warming for the earth's climates (Schleussner et al., 2016); since then, the threshold of 2°C warming had become a common standard and has been accepted by parties of the United Nations Framework Convention on Climate Change (UNFCCC, 2012). Representative of many countries attending the 2015 climate conference in Paris signed an agreement to set a goal of limiting global warming to less than 2°C when compared with preindustrial era (UNFCCC, 2015). However, the threshold of 2°C warming is not a scientific prediction (Jaeger & Jaeger, 2011), and researchers need to conduct in‐depth discussions related to how terrestrial ecosystems would respond to the climatic warming exceeding 2°C. At present, the average global temperature has increased almost 1°C since preindustrial times with the large regional difference in magnitude of warming (IPCC, 2014). The response of different ecosystems to different magnitudes of warming is far from clear, especially in China. This may hinder our future ability to manage the ecosystems in response to climatic warming in different regions.

Net primary productivity (NPP) represents the production of gross photosynthesis minus autotrophic respiration and is considered as a critical indicator for researchers who analyze the effects of climate change on terrestrial ecosystems (Ito, 2011). Quantifying the interannual variability in NPP would help us to understand the terrestrial carbon dynamics and underlying mechanisms in response to climate change (Twine & Kucharik, 2009). Numerous studies have demonstrated that warming can stimulate plant growth and carbon uptake (Delpierre et al., 2009; Oberbauer et al., 2007; Sullivan, Arens, Chinmner, & Welker, 2008; Wu, Dijkstra, Koch, Peñuelas, & Hungate, 2011). However, increased air temperature also stimulates autotrophic respiration in plants (Burton, Melillo, & Frey, 2008; Heimann & Reichstein, 2008; Knorr, Prentice, House, & Holland, 2005). Therefore, our knowledge of how NPP might respond to different magnitudes of warming is far from clear (Niu et al., 2008; Wu et al., 2011), because this response represents an integrated effect of changes in temperature and water status on photosynthesis and respiration (Angert et al., 2005; Ciais et al., 2005; Kang, Kimball, & Runing, 2006; Sullivan et al., 2008). The effects of warming on NPP will be either enhanced or weakened, depending on whether precipitation is decreasing or increasing correspondingly (Chen, van der Werf, de Jeu, Wang, & Dolman, 2013; Kang et al., 2006; Wu et al., 2011). In addition, site experiments have demonstrated that the effects of temperature increase and altered precipitation vary widely and are highly dependent on the ecosystem types and climate zones involved (Davi et al., 2006; Nemani et al., 2003; Niu et al., 2008; Wu et al., 2011). In the middle and high latitudes of the Northern Hemisphere, plant growth is mainly limited by temperature (Schwartz, Ahas, & Aasa, 2006; Wang et al., 2011), and recent climatic warming has enhanced ecosystem productivity and carbon uptake (Delpierre et al., 2009; Deng & Chen, 2011; Nemani et al., 2003; Potter et al., 2003). While in arid and semiarid areas, the ecosystem productivity in response to warming climate is mainly mediated by precipitation (Fang, Piao, Tang, Peng, & Ji, 2001; Knapp et al., 2002; Mitchell & Csillag, 2001; Niu et al., 2008).

The complex topography of China contributes to producing very diverse ecosystems, such as with the effect of the uplift of the Tibetan Plateau and the varied climate regimes from the East Asian monsoons to the western arid (Ni, 2011). These very special environmental characteristics make China a region that is particularly vulnerable to climate change (Ni, 2011), sparking concerns over the response of Chinese terrestrial ecosystems to climate change (Cao et al., 2003; Ju, Chen, Harvey, & Wang, 2007; Mu, Zhao, Running, Liu, & Tian, 2008; Peng & Apps, 1997; ). Some studies have shown that China's terrestrial NPP has increased in response to increases in temperature, altered precipitation, and elevated CO_2_ concentrations (Cao et al., 2003; Fang et al., 2003). However, responses of NPP to climate change could be remarkably diverse because of the high level of land surface heterogeneity in China and regional differences in climate change. Understanding how terrestrial NPP responds to historical warming trends and altered precipitation is very important for researchers tasked with predicting the effects of future climate change (Wang et al., 2011).

Here, we used the Carbon Exchange between Vegetation, Soil and Atmosphere (CEVSA), a process‐based model, to quantify the effects of warming on NPP in China during 1961–2010. Our main objectives were to clarify (1) whether there is a temperature threshold above which NPP no longer increases with warming; (2) whether changes in precipitation modify the response of NPP to warming; (3) if a temperature threshold exists, whether it differs among ecoregions and the effects of altered precipitation.

## 2 EXPERIMENTAL SECTION

### 2.1 The CEVSA model

The CEVSA model simulates carbon synthesis as well as water and energy exchange among vegetation, soil, and atmosphere and also models the interactions between ecosystem and environmental conditions. Detailed information on model structure and algorithms can be found in our previous publications (Cao, Prince, & Shugart, 2002; Cao & Woodward, 1998a,b; Cao et al., 2003; Gu et al., 2007; Tao et al., 2007). In CEVSA, temperature influences the photosynthesis, respiration, and stomatal behaviors. Soil water content, which is determined by the difference between precipitation and evapotranspiration in the soil, influences the ecophysiological processes by affecting the stomatal conductance.

### 2.2 Data sources

The meteorological datasets used to drive the CEVSA model include mean air temperature, precipitation, relative humidity, and cloud cover at a 10‐day time‐step. All meteorological datasets covering from 1954 to 2010 were provided by the National Meteorological Information Center of China, including 756 meteorological stations scattered across China. A 0.1° × 0.1° gridded meteorological dataset were obtained from the interpolation of the station observation data using ANUSPLINE (Hutchinson, 1989). Annual atmospheric CO_2_ concentrations were downloaded from the CO_2_• earth website (https://www.co2.earth/). The Chinese soil texture classification system was adopted, and the 1:14,000,000 soil texture map of China (The Institute of Soil Science, 1986) was digitized. The vegetation distribution map was derived from 1 km resolution Global Land Cover 2000 database (European Commission, Joint Research Centre, 2003). In order to be consistent with other input data, we aggregated the GLC2000 to obtain the 10 km land cover data by using ArcInfo 10.2. Each grid cell in the new vegetation map was assigned the value of land cover type that had the largest fraction in a 10 km grid cell.

Validation data came from three sources: (1) the recalculated National Forest Inventory (NFI) dataset (Luo, 1996; Wang, Zhou, Jiang, & Yang, 2001; Zhao & Zhou, 2004), (2) aboveground net primary productivity (ANPP) observed in Chinese grassland (Guo et al., 2012; Hu et al., 2010), and (3) cropland NPP recalculated based on agricultural statistical data. The recalculation of ANPP to NPP involves parameters such as ratio of aboveground and belowground biomass, turnover, etc. (Fan et al., 2008, 2009). Errors within all parameters would be propagated into the estimation of NPP. Therefore, we compared the observed ANPP with modeled NPP directly. County agricultural statistical data include planting area and yield of main food crops. The yield can be converted into NPP based on the water content of grain and a harvest index (Lobell, Hicke, Asner, Field, & Los, 2002; Yan, Liu, & Cao, 2007), and these data can be used to validate the regional simulation of ecosystem models. Here, for the purpose of validation, the crop NPP was estimated using Equation (1) based on yield data collected from county agricultural statistical database (Chen, 2014):

(1) $$\text{NPP}=\sum_{i=1}^{n}\frac{\frac{Y_{i}\times \left(1-{\mathrm{MC}}_{i}\right)\times 0.45\frac{\mathrm{gC}}{g}}{{\mathrm{HI}}_{i}\times 0.9}}{\sum_{i=1}^{n}A_{i}}$$

where *Y*_*i*_ is yield of crop *i*; MC_*i*_ is grain water content of crop *i*; HI_*i*_ is harvest index of crop *i*; and *A*_*i*_ is planting area of crop *i*.

We selected site‐level data that included all forest types and herbaceous covers distributed across whole China, while cropland NPP data included statistical data from more than 1,200 counties. The main time intervals of recalculated National Forest Inventory datasets were from 1980s to 1990s. The time intervals of ANPP in grassland covered from 1984 to 2005. The time of the collection cropland NPP data was 2005.

### 2.3 Model simulations

The CEVSA model was run at a 0.1° × 0.1° spatial resolution for the entire area of China with a 10‐day time‐step. First, we ran the model by using a 30‐year averaged climatic data (1954–1983) and a fixed CO_2_ concentration level in 1954 until the model reached equilibrium status and the initial state parameters were obtained. Then, the simulation was conducted using time‐variant climate and atmospheric CO_2_ data for the period 1954–2010. In addition to including the all combined simulation data such as climate change and elevated CO_2_ concentration, we also conducted three single‐factor simulations to reveal the relative effects of temperature, precipitation, and CO_2_ concentration on long trend of NPP. These three simulations were completed using (1) only air temperature change data; (2) only precipitation change data; and (3) only CO_2_ concentration change data. The modeling results from 1961 to 2010 were used in our analyses. The run from 1954 to 1960 was designed to eliminate the effect of initial status on simulations for the period 1961–2010 (Cao et al., 2002).

### 2.4 Trend and statistical analysis

We applied a least‐squares linear regression model to determine trends in temperature, precipitation, and NPP during the period 1961–2010 using Equation (2):

(2) Y=a+bX+e

where *Y* is the mean annual temperature (MAT), mean annual precipitation (MAP), or NPP; *X* is the year; *a* is the intercept; *b* is the slope, which represents the trend of variables during last 50 years and indicates the absolute change per year; and *e* is the residual error. Because the study period comprises 50 years, the total change (TC) of MAT, MAP, and NPP was calculated by multiplying the slope *b* by 50 years, and the percentage change (PC) as TC divided by the 50‐year average value of the parameters (temperature, precipitation, or NPP). All significant tests for trend analyses are *t* test.

## 3 RESULTS AND DISCUSSION

### 3.1 Model validation

We have validated the CEVSA model against multiscale observations/measurements in previous studies (Gu et al., 2007; Tao et al., 2007). In the present study, we compared the CEVSA‐estimated average NPP with those from process‐based as well as remote sensing models. Gao et al. (2012) summarized 36 estimations from these two kinds of models in China during 1980–2000, and the results showed that NPP in China was 1.43–4.60 Pg C/year. The CEVSA‐estimated NPP during the 1980–2000 was 3.83 Pg C/year and fell within the reasonable range.

**Figure 1:**
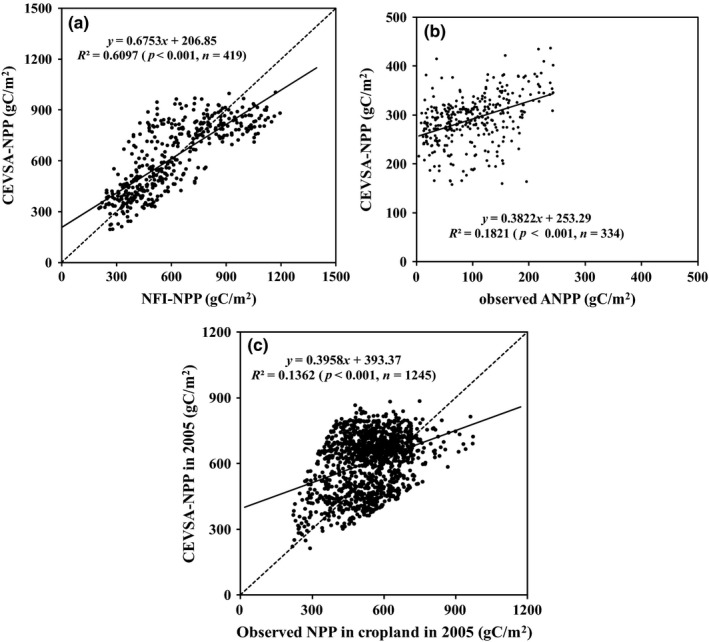
Comparison between the Carbon Exchange between Vegetation, Soil and Atmosphere systems (CEVSA) estimated net primary productivity (NPP) with field measurements. (a) The measured NPP data for 420 National Forest Inventory plots derived from recalculated datasets from other research, which include all types of forests across all of China. (b) The measured aboveground net primary productivity (ANPP) data for 335 grassland observation sites derived from recalculated dataset from other research, which was distributed across western China. (c) The NPP of cropland estimated based on statistical data for 1,246 counties. The solid line shows the linear regression fitting curve, and the dotted line is 1:1 curve

The CEVSA model simulation explained the temporal and spatial variations of observed NPP well (all *p* values were lower than .001), but there were still errors in the simulations (Figure 1). The errors came from both the model simulation and field observations. First, the spatial resolutions of model simulation and site observations were not consistent; therefore, the soil and climate data used as the model input would have large differences with the observation sites at the same time. The spatial resolution of model simulation was 0.1° × 0.1°, and the sizes of forest and grassland investigation plots were 10 m × 10 m and from 1 m × 1 m to 2 m × 2 m, respectively. The recalculated NPP in cropland had been sourced from the county level statistical data. Second, the CEVSA model does not consider the effects of all kinds of natural and human disturbance, such as nitrogen deposition, fire, harvest, management, on ecosystems. For example, the CEVSA model does not quantify the management in cropland, including irrigation, fertilization, and farming system, so the model may underestimate NPP in some areas, especially in irrigated cropland. Third, some errors were come from the recalculation of observation and statistical data. All field NPP used as validation data were not measured directly. It was calculated based on variables measured directly, such as tree height, diameter at breast height, biomass, and statistical grain yield. All the calculations would bring errors into the validated data.

### 3.2 The responses of total NPP to different magnitudes of climatic warming and the effects of changing precipitation

**Figure 2:**
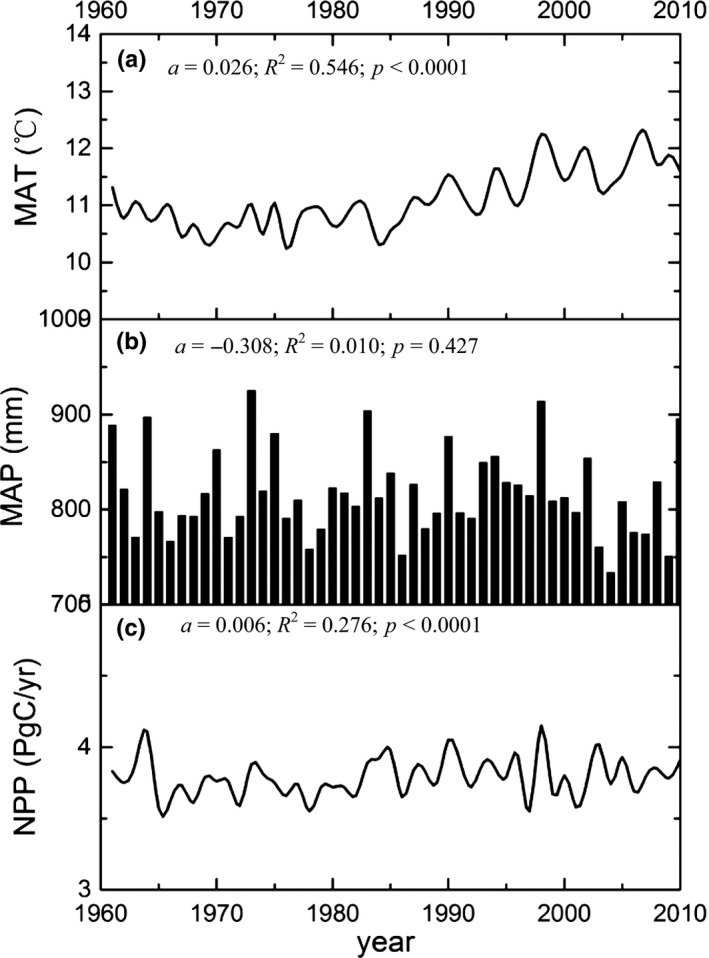
Interannual variations in mean annual temperature (MAT) (a), mean annual precipitation (MAP) (b), and net primary productivity (NPP) (c) during 1961–2010. The letter *a* represents the slope of linear regression, *R* ^2^ is determination coefficient, and *p* indicates significant factor of the *t* test

**Figure 3:**
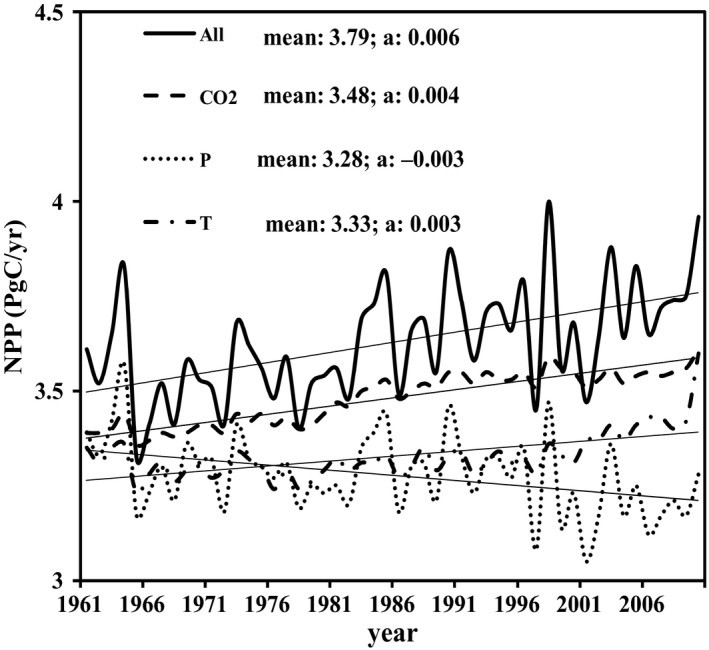
The experimental simulations of the Carbon Exchange between Vegetation, Soil and Atmosphere system (CEVSA) model with all combined factors (All), only CO_2_ concentration (CO2), only precipitation (*P*), and only temperature (*T*)

Warming resulted in an increase in NPP in China with or without the effects of CO_2_ fertilization, while decreasing precipitation resulted in a decline in NPP (Figures 2 and 3). During 1961–2010, the MAT in China showed a significant increasing trend (*p* < .001, *t* test) (Figure 2a), with a temperature change of 0.026°C/year and a total temperature change of 1.30°C over 50 years (Figure 2a). The MAP in China had a slight but insignificant decreasing trend during the study period (Figure 2b). Total NPP in China showed an increasing trend during 1961–2010, with an increase in 0.006 Pg C/year (Figure 2c). The warming resulted in an increase in 0.003 Pg C/year without the effect of CO_2_ fertilization. The decreased precipitation resulted in a decline in NPP when temperature and CO_2_ fertilization were not included.

The percentage change of NPP increased with increasing magnitude of warming (Table 1). The largest increase in NPP was found in regions where temperature increased 1–2°C regardless of any increase or decrease in precipitation, which contributed more than 64% of the total increase in NPP. A relatively high increase in NPP occurred in regions with increasing MAP when compared with regions experiencing a decrease in MAP. This indicates that negative effects of decreased precipitation surpassed the stimulation of warming on NPP; however, a decrease in precipitation did not change the variation in the increase in NPP with an increased magnitude of warming. The percentage change in NPP decreased when warming exceeded 2°C. This demonstrates that the different responses of plant photosynthesis and respiration to rising temperature may result in the transitional change of NPP when warming exceeds a certain threshold. Moreover, warming may result in enhanced evapotranspiration and a reduction in soil moisture, thereby exacerbating the water stress on ecosystem productivity as influenced by rising temperature.

**Table 1:** Percentage change (PC) in net primary productivity (NPP) for different magnitudes and trends in mean annual temperature (MAT) and precipitation (MAP)

Change in MAT	PC of NPP (%)	PC of NPP for increasing MAP (%)	PC of NPP for decreasing MAP (%)	Percentage of the total area (%)
Average	7.87	9.11	6.89	–
≤0°C	5.23	8.28	−0.01	7.37
0–1°C	6.61	7.97	5.02	14.4
1–2°C	9.83	10.26	9.50	52.6
>2°C	5.40	7.68	4.10	25.6

The second assessment report of the IPCC (Houghton et al., 1996) integrated the research results of more than 1,000 scientists and pointed out that the risk of serious negative effects from climate change would increase significantly if the average global temperature increased over 2°C above preindustrial levels. In 1996, the European Union initially proposed 2°C as a red line for climatic warming that should not be exceeded (European Environment Agency, 1996), and climate change scientists of the European Union released “The 2°C target” evaluation report in 2008 (EU Climate Change Expert Group, 2008). This report pointed out that human beings might not suffer from the expected effects of climate change on the economy, society, and environment if the increase in the average global temperature was not held below 2°C (Jaeger & Jaeger, 2011). No evidence supports the concept that human society will be able to adapt to the climate change if the increase in average global temperature is above 3°C or 4°C (Knutti, Rogelj, Sedláček, & Fischer, 2016; Kypreos & Magné, 2013). However, in the present study, the total NPP is not expected to decrease even if the temperature increased by over 2°C, although the increasing trend of NPP decreased conspicuously. Therefore, 2°C did not become the threshold of limiting ecosystem functions in whole China even though the rate of increase in NPP declined at higher temperature.

### 3.3 The different responses of NPP to warming in different ecoregions and the effects of altered precipitation

**Figure 4:**
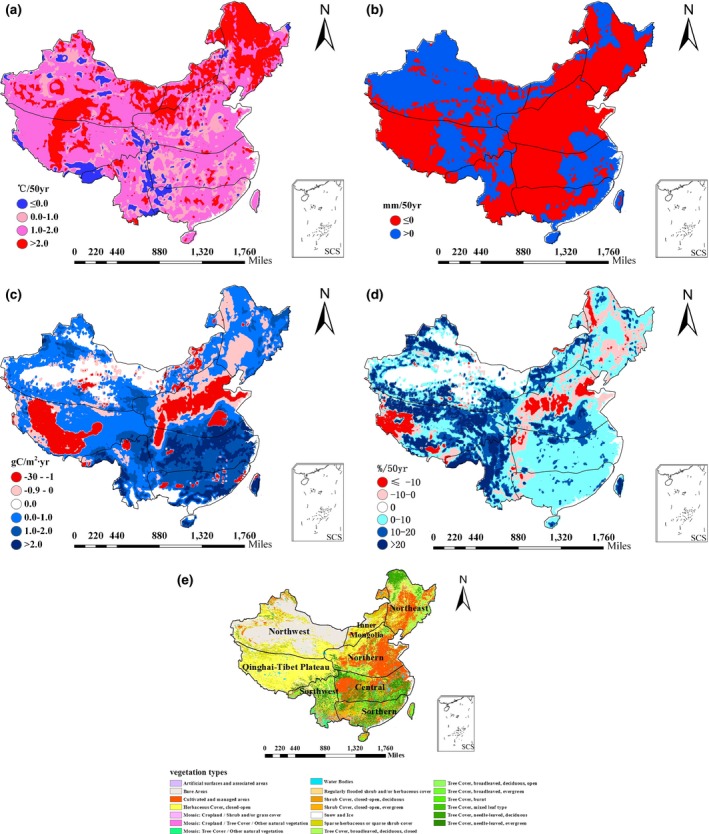
The spatial distribution of (a) change in mean annual temperature (MAT) over 50 years in unit of °C/50 years; (b) change in mean annual precipitation (MAP) over 50 years in mm/50 years; (c) trend in net primary productivity (NPP) during 1961–2010; (d) percentage change of net primary productivity (NPP) over 50 years; (e) vegetation map and ecoregions in China. SCS denotes “South China Sea.” Northeast, North, Inner Mongolia, Central, South, Southwest, Northwest, and Qinghai–Tibet Plateau represent the different ecoregions in China

The total NPP in China has exhibited an increasing trend with obvious spatial variations (Table 2, Figure 4). Over the past 50 years, North received the largest decrease in precipitation, and Northeast and Inner Mongolia experienced the largest increase in air temperature (Table 2, Figure 4a and b). The decrease in NPP mainly occurred in North, Northeast, and the western part of Central China. Our research confirmed the results of Piao et al. (2011) and Zhao and Running (2010), which also found a negative trend for NPP in Northeast and North China. The western part of Southwest, Central and South China have the largest increases in NPP (Figure 4c). Piao et al. (2011) estimated that the increase in NPP in Southern China would be greater than 3 g C m^−2^ year^−1^, and our estimation was greater than 2 g C m^−2^ year^−1^. The largest percentage change in NPP occurred in the mountainous areas of Northwest, Qinghai–Tibet Plateau and the western part of Southwest (Figure 4d).

**Table 2:** Trends in net primary productivity (NPP; g C m^−2^ year^−1^) for different magnitudes of warming (*T*) in regions with different changes in mean annual precipitation (MAP)

Region	MAT	MAP	Trends in MAT	Trends in MAP	Trends in NPP mean	0°C < *T* ≤ 1°C			1°C < *T* ≤ 2°C			*T* > 2°C		
						Mean	MAP increase	MAP decrease	Mean	MAP increase	MAP decrease	Mean	MAP increase	MAP decrease
Northeast	2.0	519	2.12	−27.13	0.12	0.66	0.59	0.74	0.26	0.39	0.21	−0.02	0.28	−0.11
North	10.1	603	1.34	−82.14	0.15	0.83	1.95	0.67	0.01	1.95	−0.14	−0.52	–	−0.52
Inner Mongolia	3.7	288	1.94	−26.90	0.43	1.03	1.08	0.64	0.35	0.72	0.24	0.34	0.80	0.30
Central	15.8	1369	1.35	13.36	1.34	1.19	1.14	1.32	1.39	1.35	1.45	1.37	1.31	1.42
South	19.7	1636	1.21	29.27	1.45	1.28	1.30	1.25	1.62	1.50	1.72	1.47	1.54	1.45
Southwest	13.3	1073	0.73	−60.43	0.97	0.51	0.70	0.48	1.92	2.48	1.70	1.19	1.28	1.16
Qinghai–Tibet Plateau	−1.9	404	1.23	−14.85	0.65	−0.08	0.26	−1.23	0.72	0.75	0.71	0.76	0.50	0.93
Northwest	5.2	143	1.54	20.48	0.29	0.25	0.26	–	0.29	0.32	0.23	0.31	0.31	0.31

Units: MAT and *T*, temperature in °C; trends in NPP in g C m^−2^ year^−1^; MAP in mm; –, no data.

In different ecoregions, the responses of NPP to warming showed three types of patterns (Table 2). (1) In Northeast and North China as well as Inner Mongolia, the increasing trend of NPP decreased in response to warming, especially when precipitation decreased. We also noticed that the increasing trend of NPP decreased even when precipitation increased, while decreasing precipitation merely reduced the rate of increase for NPP. (2) In Central, South, and Southwest China, warming stimulated an increase in NPP until the increase in temperature exceeded 2°C regardless of any increase or decrease in precipitation, which was compared with the trend for NPP in whole China. In these ecoregions, the NPP had an even higher rate of increase when precipitation decreased. (3) On the Qinghai–Tibet Plateau and in Northwest China, the positive trend of NPP increased with warming. Increased precipitation contributed to an increase in NPP (Dai, Zhang, Wang, Guo, & Wang, 2010; Dan, Ji, & Ma, 2007). All these indicate that ecosystems have different responses to changes in increased temperature and precipitation in different regions and climatic zones. Therefore, climate change research should not promote the use of a single threshold or a target for warming in a global context. A better approach would be to establish regional thresholds for warming based on the different responses of various ecosystems to warming and changes in precipitation in different regions and climatic zones. Therefore, models such as CEVSA could be a useful tool to determine reasonable regional warming thresholds.

Falloon et al. (2007) suggested that while the pattern of warming is widespread in all areas, regional disparities between warming and NPP may be related to variations in precipitation, which shows strong variations regionally. Generally, a decrease in precipitation did not influence the response of an increase in NPP to warming (Table 1). However, the change in precipitation played different roles in responses of NPP to warming in different ecoregions (Table 2).

Temperature had been considered to be the principal limiting factor on vegetation growth in temperate and relatively cold areas (Nemani et al., 2003). Our results suggest that warming does not always result in an increase in NPP, even in areas where temperature was considered as the primary controlling factor for NPP. Recent research (Boisvenue & Running, 2006; Nemani et al., 2003; Running et al., 2004) and our results have shown that the vegetation growth across the entire northern China was limited by precipitation. Water availability plays a dominant role in plant growth and net ecosystem productivity in some regions (Niu et al., 2008; Potts, Huxman, Enquist, Weltzin, & Williams, 2006; Weltzin et al., 2003). In Northeast and North China as well as Inner Mongolia, NPP showed a decreasing trend in regions where temperature increased by more than 1°C while precipitation decreased (Table 2). Obviously, a combination of warming and decreased precipitation tend to be colimiting on trends of NPP in northern China, while warming enhanced the stress of water deficit induced by decreased precipitation on plant growth. In addition, the water shortage induced by warming could also reduce the increasing trend of NPP even when precipitation increased (Wu et al., 2011).

In southern China, which receives the greatest amount of precipitation (more than 1,000 mm), decreased precipitation does not necessarily result a decline in NPP and may even exacerbate the increase in NPP (Table 2 and Figure 4c). The stimulation of decreased precipitation on NPP in southern China may be originated from an increase in radiation induced by decreased precipitation; this occurred because the vegetation growth in southern China is limited by photosynthetically active radiation received by the canopy (Nemani et al., 2003). Photosynthetically active radiation was calculated using the CEVSA model, which was determined by cloud cover; therefore, it was influenced by the change of precipitation. In fact, the change of surface solar radiation was influenced by many factors, such as sunshine duration, cloud cover, the presence of aerosols. Various studies suggested a decrease in surface solar radiation has occurred in southern China during the past decades, with a partial recovery more recently (Wang, Huang, & Zhang, 2009; Zheng, Guan, Cai, Wu, & Liu, 2011); however, the level of diffuse photosynthetically active radiation increased in all of China, especially in southern China (Ren, He, Zhang, & Yu, 2014).

In Northwest China, the increase in precipitation has been associated with the largest percentage change in NPP. Some evidence from satellite data and other sources has also confirmed the positive trends in NPP during the past 30 years in the northwestern part of China (Dai et al., 2010; Dan et al., 2007). Previous studies also confirmed the climate evolved from warm‐dry to warm‐wet in northwest China (Liu, Feng, Ma, & Wei, 2009; Shi et al., 2003). Increased precipitation stimulated plant growth which was limited mainly by water scarcity in northwest China, so the vegetation index was obviously increased and the number of days experiencing sand‐dust storm decreased (Shi et al., 2003).

### 3.4 Uncertainty analysis and future research needs

Uncertainties are inevitable in modeling regional NPP and its response to global changes (Ito, 2011). The sources of uncertainty derive mainly from three aspects: (1) *the input data, parameters, and the spatial resolution, including meteorological data, land use/land cover dataset, and soil data*. For example, the sparse distribution of meteorological stations in western China, especially on Qinghai–Tibet Plateau, may introduce large errors into the analysis. Thus, the spatial and temporal variations in temperature and precipitation should be closely examined and validated, and more accurately interpolated meteorological data should be applied to analyze the effects of climate change on NPP in these regions. (2) *The representation of carbon and water cycle processes in the model*. For example, cultivated areas serve as the main land use in North China (Figure 4e). However, the CEVSA model did not include the effects of human management on NPP, such as irrigation, fertilization, as well as the change in the variety of crops cultivated on cropland. Therefore, we may have underestimated the NPP in North China. Another example involves plant growth in most areas of western China where water is supplied mainly by runoff instead of precipitation. However, the model simulates precipitation as providing water for plant growth instead of runoff; thus, the NPP in these regions may also be underestimated. A combination of hydrological and ecosystem models could better simulate the effects of water resources on the carbon cycle in these regions in the future. (3) *In the present study, we did not consider disturbances (e.g., fire, grazing) and other environmental factors* (e.g., nutrient availability, nitrogen deposition, ozone pollution). Disturbances may also bring uncertainties into the simulated results. An accurate estimation of the carbon budget of the terrestrial ecosystem is important during the evaluation of the effect and relative contributions of multiple environmental factors on productivity and carbon accumulation in terrestrial ecosystems (Ren et al., 2007; Tian et al., 2011; Wang et al., 2011). Incorporating the effects of all natural and anthropogenic factors into the model simulation will require greater effort related to the clarification and quantification of all these processes in the models. With the further development of the ecosystem model, it is necessary to analyze the effects of all these factors on ecosystem and their relative contributions to the carbon cycle. In the future, we may identify the uncertainty range of modeled carbon fluxes at a regional scale by using data‐assimilation techniques (Cao, Yu, Liu, & Li, 2005; Ito, 2011; Rayner et al., 2005; ; Zhang et al., 2012). In addition, the multiple model intercomparison provides another method that can be used to evaluate the uncertainties in model simulations of NPP in response to climate change and variability.

In the present study, we only examined the responses of NPP to warming and altered precipitation in different ecoregions. We also need to understand the mechanisms that control the different responses of NPP to warming and changing precipitation by conducting simulation experiments as well as by making observations in different ecosystems and climate zones. Nevertheless, previous studies have demonstrated the stimulation of warming on productivity (Delpierre et al., 2009; Oberbauer et al., 2007; Sullivan et al., 2008; Wu et al., 2011) and the varied impact of altered precipitation (Chen et al., 2013; Kang et al., 2006; Wu et al., 2011). The different responses of plant growth to warming and altered precipitation as well as the related mechanisms in different ecosystems are far from clear. Scientists also need to determine the nature of the threshold of warming at a point when NPP changes from having a positive response to warming to a negative one. In addition, changes in many factors, such as elevated atmospheric CO_2_ concentration, nutrient availability (especially the rising nitrogen and sulfur deposition), human management, would influence the responses of ecosystems to climate change. Clarifying the different responses of ecosystems to climate change under the influence of all environmental factors, disturbances, and human management is needed. All these efforts will contribute to determining whether a threshold of warming exists and what is the nature of threshold in different regions.

## 4 CONCLUSIONS

As for China as a whole, no one temperature threshold exists above which NPP no longer increases with warming during past climate warming; however, we found that the positive trend of NPP decreased when warming exceeded 2°C regardless of whether precipitation increased or decreased. This result should be further verified by the integration of simulations by other models and through the analysis of remote sensing observational data. The responses of NPP to warming and corresponding precipitation change varied in different ecoregions. All ecoregions showed three patterns of response to warming that were determined by the conditions in the local ecoregional climate and changes in precipitation. It will be better to establish a regional threshold for warming based on the different responses of various ecosystems to warming and to changes in precipitation in different regions and climatic zones. In addition, we should focus on how the different types of ecosystem in different climatic zones respond to climate change and how the ecosystems adapt to the change, rather than setting a fixed threshold for all regions of China regions or on a global scale.

For accurate estimation of terrestrial ecosystem productivity, it is important to represent the known biogeochemical processes and their mechanisms, as well as the effects of interactions among multiple factors on the carbon cycle in ecosystem models. Moreover, we should try to reveal the different responses of plant growth to warming and altered precipitation patterns as well as the mechanisms involved in different ecosystems. The threshold of warming that occurs when NPP changes from a positive response to warming to a negative one should also be determined.

## CONFLICTS OF INTEREST

The authors declare no conflict of interest.
